# Self-reported responsiveness to direct-to-consumer drug advertising and medication use: results of a national survey

**DOI:** 10.1186/1472-6963-11-232

**Published:** 2011-09-23

**Authors:** Nicholas J Dieringer, Lisa Kukkamma, Grant W Somes, Ronald I Shorr

**Affiliations:** 1 Department of Medical Education, Methodist Healthcare, 1265 Union Avenue, Memphis, TN 38104, USA; 2 GRECC (182) NF/SG Veterans Health System 1601 SW Archer Road, Gainesville, FL 32608, USA; 3 Department of Preventive Medicine, University of Tennessee Health Science Center, Memphis, TN 3810, USA; 4 Department of Aging and Geriatric Research, University of Florida, Gainesville, FL, USA

**Keywords:** Advertising, medication use, consumerism, prescription medication, non-prescription medication

## Abstract

**Background:**

Direct-to-consumer (DTC) marketing of pharmaceuticals is controversial, yet effective. Little is known relating patterns of medication use to patient responsiveness to DTC.

**Methods:**

We conducted a secondary analysis of data collected in national telephone survey on knowledge of and attitudes toward DTC advertisements. The survey of 1081 U.S. adults (response rate = 65%) was conducted by the Food and Drug Administration (FDA). Responsiveness to DTC was defined as an affirmative response to the item: "Has an advertisement for a prescription drug ever caused you to ask a doctor about a medical condition or illness of your own that you had not talked to a doctor about before?" Patients reported number of prescription and over-the-counter (OTC) medicines taken as well as demographic and personal health information.

**Results:**

Of 771 respondents who met study criteria, 195 (25%) were responsive to DTC. Only 7% respondents taking no prescription were responsive, whereas 45% of respondents taking 5 or more prescription medications were responsive. This trend remained significant (p trend .0009) even when controlling for age, gender, race, educational attainment, income, self-reported health status, and whether respondents "liked" DTC advertising. There was no relationship between the number of OTC medications taken and the propensity to discuss health-related problems in response to DTC advertisements (p = .4).

**Conclusion:**

There is a strong cross-sectional relationship between the number of prescription, but not OTC, drugs used and responsiveness to DTC advertising. Although this relationship could be explained by physician compliance with patient requests for medications, it is also plausible that DTC advertisements have a particular appeal to patients prone to taking multiple medications. Outpatients motivated to discuss medical conditions based on their exposure to DTC advertising may require a careful medication history to evaluate for therapeutic duplication or overmedication.

## Background

Considerable controversy surrounds the practice of direct-to-consumer (DTC) marketing of pharmaceuticals. While some argue that DTC advertising serves as an educational resource for patients,[1] others say that DTC advertising contributes to the medicalization of trivial ailments, and leads to overuse and misuse of pharmaceuticals[2]. Since initially allowed by the Food and Drug Administration in 1985,[3] spending on DTC advertising grew steadily over the following decade. DTC advertising spending accelerated rapidly after 1997, when the Food and Drug Administration provided draft guidance which described how sponsors could disseminate information on drugs and biological products using radio and television [4,5]. Pharmaceutical industry expenditures for DTC advertising increased 330% between 1996 and 2005. In 2005, $4.2 billion was spent on DTC advertising, which accounted for 14% of all promotional spending for prescription drugs[6]. Expenditures have decreased (in absolute terms) since 2007 following a peak of $5.4 billion in 2006 [7].

Although it is difficult to find data on the effectiveness of DTC advertising for individual drugs, 10-35% of survey respondents say they have discussed medical conditions or medications with their physicians as a result of DTC advertising[8-10]. Furthermore, patients who specifically request medications during an office visit are much more likely to receive them than those who do not[9,11-13].

While much has been written regarding consumer and physician attitudes toward DTC advertising, less is known relating patient characteristics, notably medication use, and responsiveness to DTC advertising. One survey found that responders to DTC advertising were more likely to be nonwhite, have lower educational attainment and lower income,[10] whereas Bell found that women, as well as persons with a positive attitude towards DTC advertising, in poorer self-reported health, and with better self-reported insurance coverage of medications were more likely to respond to DTC advertising[9]. This survey also found that persons using prescription drugs at the time of the interview were more likely to be influenced by DTC advertising. To further investigate the relationship between self-reported medication use and responsiveness to DTC advertising, we conducted a secondary analysis of data obtained in an FDA survey of exposure to, perceptions of, and attitudes toward DTC advertising.

## Methods

### Data Source

The data come from a national telephone survey of adults in the U.S., investigating the demographic characteristics and attitudinal effects of DTC prescription drug advertising of survey respondents[5]. The survey was conducted by a professional research firm for the Food and Drug Administration between April and July 1999 to examine attitudes and influence of DTC advertising of prescription drugs upon the healthcare experience. The target population for the survey was English speaking consumers aged 18 or older.

Respondents were contacted using random digit dialing methodology and several steps were taken to optimize the response rate. Interviews were scheduled for varying times of the day in an effort to locate potential respondents at a convenient time. Respondents were allowed to schedule "call back" appointments to complete the survey at a more convenient time. Unlimited call backs (more than 40) were utilized for phone numbers that did not initially yield a respondent contact. Respondents who initially refused to participate, or terminated the interview before completion were contacted by a mailed follow-up letter as well as two follow-up telephone solicitations.

### Study Population

1081 persons responded to the survey representing a response rate of 65% (eligible respondents who completed survey plus ineligible respondents divided by the total contacted minus bad telephone numbers). From these, 310 persons who had not been exposed to DTC prescription drug marketing in the three months prior to the survey, and therefore did not answer the question of interest, were excluded. Table 1 describes those persons excluded from the analysis. Thus our study population included 771 persons.

**Table 1:** Characteristics of Respondents Not Exposed to DTCA, Excluded From Study

Characteristic	Prevalence
**Age**	
Less than 65 years	72.0
65 years or older	28.0
**Gender**	
Male	35.2
Female	64.8
**Race**	
White	66.5
Non-white	33.5
**Marital Status**	
Married	49.7
Single, widowed, divorced, other	50.3
**Educational Attainment**	
College graduate or higher	23.2
Some college or less	76.8
**Annual Income**	
Less than $35,000	57.9
$35,000 or more	42.1
**Time since last visit to physician**	
Within one month	40.7
More than one month	59.3
**Self-reported health**	
Excellent	17.8
Very Good	29.1
Good	27.2
Fair	18.1
Poor	7.8
**Mean # of Rx drugs in past 6 months**	2 (S.D. 1.6)
**Mean # of OTC drugs in past 6 months**	1.2 (S.D. 1.2)

n = 310

### Main Measures

"Responsiveness to DTC drug marketing" was defined as an affirmative response to the survey item: "Has an advertisement for a prescription drug ever caused you to ask your doctor about a medical condition or illness of your own that you had not talked to a doctor about before?" Prescription and OTC drug use was determined using the self-reported number of prescription drugs, and OTC drugs used within the six month period prior to the survey.

### Covariates

Covariates were also determined from the survey. These included gender, race, income, marital status, date of last physician visit, self-reported health, self-reported knowledge about health and medications, and attitude toward DTC drug advertisements. Self-reported health was assessed with a single item using a five point scale ranging from 1 ("excellent" health) to 5 ("poor" health). Self-reported knowledge about health and medications was assessed with a single item using a four point scale ranging from 1 ("extremely" knowledgeable) to 5 ("not at all" knowledgeable). Attitude toward DTC drug advertisements was assessed with a single item asking how much the respondent "liked" DTC advertising with responses on a five point scale ranging from 1 ("strongly agree") to 5 ("strongly disagree").

### Statistical Analysis

We used T-test and chi-square tests for univariate analyses. For multivariate analysis, we used logistic regression controlling for covariates. These covariates included age, gender, race, educational attainment, marital status, income, self-reported health status, self-reported knowledge of medicines and health, and attitude toward DTC advertising. We performed all analyses using SAS version 9.1 (SAS Institute Inc, Cary, NC). All p-values were 2-sided and α was set at .05.

## Results

### Characteristics of the Study Sample

The average respondent's age was (mean ± s.d.) 46.1 ± 15.7 years, 64% were female, and 81% were white. Forty-two percent had completed at least a college degree and 57% were married at time of interview. On average, respondents had encountered 2.4 ± 1.3 modes of delivery of DTC prescription drug advertising (e.g. magazines, television, newspaper, etc.) in the three months prior to the interview. Respondents' mean score for how much they "liked seeing" these advertisements was 2.7 ± 1.4. The mean response for respondents' self-reported knowledge of medications and health matters in general was 2.6 ± 0.7. Similarly, mean response for respondents' self-reported health status was 2.4 ± 1.1 see Table 2.

**Table 2:** Sample Characteristics by Responsiveness to DTC Advertising

Covariate	Prevalence	Proportion responsive to DTC advertising	Multivariate association with responsiveness to DTC advertising (OR, 95% CI)
**Age**			
Less than 65 years	85.9	23.2	2.5 (1.5-4.1)
65 years or older	14.1	37.6	
**Gender**			
Male	36.1	22.2	1.0 (.7-1.5)
Female	63.8	27.3	
**Race**			
White	82.6	24.5	.7 (.4-1.0)
Non-white	17.4	31.1	
**Marital Status**			
Married	57.5	25.5	1.1 (.7-1.6)
Single, widowed, divorced, other	42.1	25.0	
**Educational Attainment**			
College graduate or higher	57.8	28.9	.7 (.4-1.0)
Some college or less	42.1	20.3	
**Annual Income**			
Less than $35,000	48.6	25.8	1.2 (.8-1.9)
$35,000 or more	51.3	24.8	
**Self-reported health**			
Excellent	23.2	13.4	1.1 (1.0-1.4)
Very Good	41.4	25.1	
Good	22.1	32.9	
Fair	11.9	28.5	
Poor	4.4	44.1	
**Time since last visit to physician**			
Within one month	59.6	27.4	.9 (.6-1.4)
More than one month	40.4	22.2	
**Self-reported knowledge about health and medications**			
Extremely knowledgeable	8.8	22.1	1.0 (.8-1.3)
Very knowledgeable	30.0	25.5	
Somewhat knowledgeable	58.2	25.4	
Not at all	3.0	26.1	
**Like seeing advertisements for prescription drugs**			
Strongly agree	20.4	41.6	1.4 (1.2-1.6)
Agree somewhat	31.0	24.4	
Neither agree or disagree	20.8	20.7	
Disagree	10.9	18.1	
Strongly disagree	16.9	15.5	

n = 771

### Relationship between Respondent Characteristics and Responsiveness to DTC Marketing

Overall, 195 (25.3%) respondents answered affirmatively to our variable of interest, indicating that they had initiated discussion of a medical problem with their physician based on DTC advertising. Compared to respondents who denied discussing medical problems with their physicians based on DTC advertising, respondents who used DTC advertising as a foundation for discussing a new health problem with their physician were older, possessed less educational attainment, and "liked" seeing DTC prescription drug advertisements. Gender, race, marital status, self-reported health status, self-reported knowledge about health and medications, and income were not significantly associated with the primary endpoint (see Table 2).

### Relationship between Self-Reported Prescription Medication Use and Responsiveness to DTC Marketing

**Figure 1:**
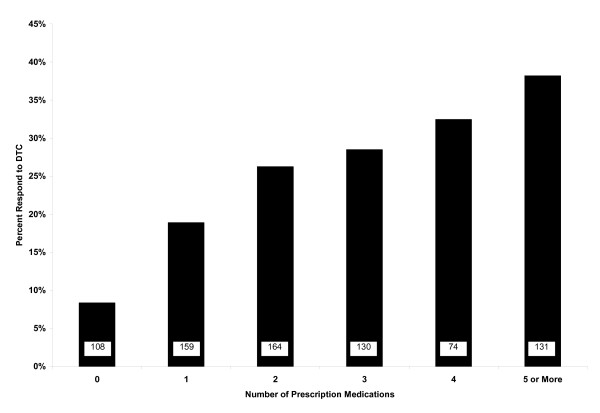
**Proportion of respondents who were responsive to direct-to-consumer (DTC) advertising, by number of self-reported prescription medications used in six months prior to study**. The number of respondents in each category is embedded in the bars.

The mean number of prescription drugs used in the six months prior to the survey was 2.4 ± 1.6. Of 108 persons using no prescription medications, 9 (8.3%) were responsive to DTC advertisements. Of 131 persons using five or more prescription medicines, 50 (38.1%) were responsive to DTC advertisements. There was a strong trend (p < .0001) in the relationship between number of prescription medications used and responsiveness to DTC advertisements. This association remained highly significant (p = .0009) even when controlling for demographics, self-reported health status, educational attainment, race, marital status, self-reported knowledge of medications and health, number of OTC medications used, income, and attitude toward DTC advertisements see Figure 1.

### Relationship between Self-Reported OTC Medication Use and Responsiveness to DTC Marketing

**Figure 2:**
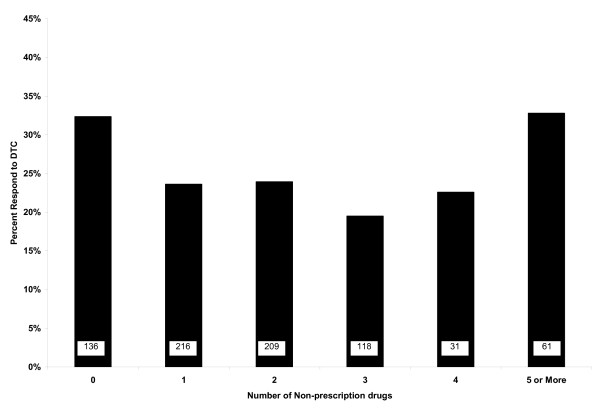
**Proportion of respondents who were responsive to direct-to-consumer (DTC) advertising, by number of self-reported non-prescription medications used in six months prior to study**. The number of respondents in each category is embedded in the bars.

The average number OTC drugs used in the six months prior to the survey was 1.8 ± 1.4. Of 136 persons using no OTC medications, 44 (32.3%) were responsive to DTC. Of 61 persons using five or more OTC medicines, 20 (32.7%) were responsive to DTC. There was no linear trend in either univariate (p = .54.) or multivariate (p = .76) relationship between number of OTC medications used and responsiveness to DTC see Figure 2.

## Discussion

Our data indicate that a cross-sectional relationship exists between number of prescription drugs used and "responsiveness" to DTC advertising. This strong linear relationship remains even when controlling for demographic and socioeconomic variables and self-reported health. Overall, 25.3% of respondents were motivated by DTC advertising to initiate discussion regarding new health problems. Among respondents taking no prescription medications, fewer than 10% were motivated by DTC advertising. Conversely, among respondents taking five or more prescription medicines, nearly 40% were motivated.

In 2002, the Government Accountability Office has identified 13 surveys, primarily for lay publications, which attempted to assess consumer behavior related DTC advertising[8]. Since that time several additional surveys have been published in the medical and scientific literature[10,14-16]. In these studies, the proportion of respondents who reported being influenced by DTC advertising to seek medical care ranged from 10.5 to 35%. Although there is some variation in how each survey defines responsiveness to DTC advertising, our finding that 25% of respondents had discussed a new medical condition with a physician based on DTC advertising is generally consistent with others.

Our results confirm those of previous studies, [10,17] which identify older age, lower educational attainment and a positive attitude towards DTC advertising as factors associated with responsiveness to DTC advertising[9,10]. In contrast to other studies, [14,18] we did not find an association between ethnicity, income or self-reported health status and DTCA.

We found that a cross-sectional relationship exists between responsiveness to DTC and self-reported use of prescription, but not OTC, drugs. Because there was a trend towards poorer self-reported health and responsiveness to DTC, is plausible that users of prescription medications represent a sicker population, and one that may be more attuned to the messages conveyed in DTC advertising. Alternatively, because DTC advertising largely focuses on prescription medications, persons who are responsive to DTC may have approached their prescribers based on information in DTC, resulting in more prescription, rather than OTC, use. Bell and colleagues found that current users of prescription drugs were more likely to be influenced by DTC advertising,[9] but did not quantify the number of prescriptions, and limited their sample to one county in California.

Our investigation confirms results of a smaller study of Minnesotans by Schommer et al. [15] that found prescription medication use to be statistically greater in respondents who were responsive to DTC advertising than those who were not. Similar to our results, no significant difference was found in the number of OTC medications used by responsive versus unresponsive patients.

The main strength of our study is that the source of data comes from a large nationally representative sample with a high response rate. Furthermore, unlike some other studies, OTC as well as prescription drug use was ascertained. Our study has several limitations. First, these data were collected in 1999. Penetration and awareness of DTC advertising, as well as types of media outlets (e.g. internet), have increased considerably in subsequent years. Despite this, however, we are encouraged by similarities in regard to attitudinal, socioeconomic and demographic predictors of susceptibility to DTC advertising demonstrated in more recent surveys, [14,15] and likewise feel that the relationship between number of prescription drugs used and susceptibility to DTC advertising should be no less true today. Second, this is a secondary analysis of FDA data originally collected to investigate attitudinal and demographic effects toward DTC advertising; thus, rigorous ascertainment of medication use was not included. Although the self-reported medication use measure is a limitation, it has been used in a previous survey of DTC advertising,[15] and has been found to be congruent with pharmacy prescription data[19,20].

The cross-sectional nature of this study does not allow for unequivocal statements regarding the direction of causality. Although this relationship could be explained by physician compliance with patient requests for medications, it is also plausible that DTC advertisements have a particular appeal to patients prone to taking multiple medications. Finally, the main measure in this study specifically asks about initiating discussion with a physician about a new medical problem and thus may not have captured those patients who use DTC advertisements to prompt discussion of previously discussed problems, or those patients who may have discontinued a medication due to information contained in DTC advertisements.

## Conclusion

There is a strong cross-sectional relationship between the number of prescription, but not OTC, drugs used and responsiveness to DTC advertising. Although this relationship could be explained by physician compliance with patient requests for medications, it is also plausible that DTC advertisements have a particular appeal to patients prone to taking multiple medications. Outpatients motivated to discuss medical conditions based on their exposure to DTC advertising may require a careful medication history to evaluate for therapeutic duplication or overmedication.

Further research needs to be performed to understand this population's eagerness to utilize prescription drugs and to determine if this eagerness transcends to utilization of other health care resources. Clearly, these DTC advertisements are powerful motivators. Perhaps they could be used to enhance evidence-based prescribing and advance public health.

## Competing interests

The authors declare that they have no competing interests.

## Authors' contributions

ND and RS conceived of the study, participated in its design, performed statistical analyses, interpreted the data, and helped draft and revise the manuscript. LC and GS participated in the analysis and interpretation of the data and helped draft and revise the manuscript. All authors read and approved the final manuscript.

## Pre-publication history

The pre-publication history for this paper can be accessed here:

http://www.biomedcentral.com/1472-6963/11/232/prepub
